# Water quality and trophic status of Lake Mariut in Egypt and its drainage water after 8-year diversion

**DOI:** 10.1007/s10661-022-10009-8

**Published:** 2022-04-28

**Authors:** Nashwa A. Shaaban

**Affiliations:** Oceanography Department, Faculty of Science, Alexandria University, Alexandria, Egypt

**Keywords:** Lake Mariut, Alexandria City, Water quality, Nutrients, Trophic status, Rehabilitation, Eutrophic lake

## Abstract

Water resource management and lake rehabilitation are global interest, to overcome the acute water scarcity facing most urban areas, especially in developing countries. Lake Mariut (LM) is an Egyptian lake that lies south of the Mediterranean Sea, and its management had a great interest in Alexandria’s future development and as a part of the environmental sustainability of the Mediterranean basin. LM consists of 4 major basins, namely, main (MB), northwest (NWB), southwest (SWB), and fishery (FB). The MB has deteriorated as it consistently received (sewage and industrial) wastewaters till 2010. This was the date of diversion of the polluting sources as a rehabilitation step. The present work is made after elapsing 8 years from the diversion and aims at monitoring and assessing the status of the water quality not only of MB but also for the other 3 basins too. This was carried out twice, one in cold winter and the other in warm summer. The parameters studied were physico-chemical including water temperature, pH, dissolved oxygen/hydrogen sulfide (DO/H_2_S), salinity, and chlorophyll *a* besides nutrient salts (nitrogen, N and phosphorus, P compounds). The results revealed that the waters of both MB and SWB basins were depleted in DO to a level difficult to sustain fish life and showed elevated levels of most nutrient salts. Trophically, all the studied basins were in eutrophic-hypereutrophic condition. As a result of pollution source diversion away from MB to be discharged in the downstream part of Umum Drain (UD), the levels of TN and TP entering Mex Bay through UD were slightly higher than the permissible limit and lower than dangerous loading. The applying of principle component analysis (PCA)-based classification approach, for obtaining the WQI on the current study, revealed that the FB, NWB, and SWB_2000_ showed a relatively good water quality when compared with MB. The depletion in the vital DO is catastrophic and needs urgent solution/s. One of the proposed solutions is the dredging of bottom spoiled organic-rich sediments. This and other suggested solutions are, however, discussed, evaluated, and presented.

**Supplementary information:**

The online version contains supplementary material available at 10.1007/s10661-022-10009-8.

## Introduction

The water quality plays a vital role in keeping both the native biota and the surrounding ecosystem healthy and productive. The recent growth in population and urbanization have a substantial effect on the local environment and cause water quality degradation. This is due to the increase in industrial, agricultural, and sewage effluents that are discharged into the aquatic environment (Lin et al., 2020). Subsequently, lake eutrophication (due to natural, cultural, or both) is a water environment challenge in the world, where the cultural eutrophication arises due to the excessive nutrients (nitrogen, N, and phosphorus, P) loading from anthropogenic origins, leading to considerable growth of aquatic flora, especially harmful algal blooms, loss of biodiversity, food-web alternations, and death of aquatic life (Xu et al., 2014). In addition, the eutrophication affects the retaining the nutrients (especially P) in the sediment (after their entrance to the lake), which led to their accumulation over time and generates P- and organic matter-enriched sediment (Song et al., 2017). Eutrophication usually coincided with water quality deterioration (Nikolai & Dzialowski, 2014), which not only reflected in continuous depletion in dissolved oxygen (DO), and pH changes of the water column of such environment (as a result of aerobic decomposition of organic matter), but also displayed in the release of P back from sediment into the overlying water (termed as internal P loading or internal fertilization). Thus, the internal P loading becomes a potentially large P source compared to external P loading, which also declines the water quality (Alhamarna & Tandyrak, 2021).

Thus, the management of the lake is essential for generating and restoring the aquatic environment suitable for growing a healthy life. Generally, there are two broad approaches concerning the water quality management of lakes: (1) the reduction of external loading (termed also as preventive, remediation, or indirect methods) and (2) the lake-internal restoration (curative, ameliorating, or direct methods) (Alhamarna & Tandyrak, 2021), noting that the former approach has the priority over the later one (Zamparas & Zacharias, 2014). This preventive approach is based on identifying the pollutants, decreasing their generation rate, and/or preventing them from entering the lake (Kasprzak et al., 2018). However, the remediation (preventive) approach of the drainage basin is convenient for shallow, polymictic lakes with short-lasting water residence time. The synchronized application of various methods will increase the efficiency of lake restoration. For example, managing and reducing the external load should be accompanied by the dredging of nutrient-rich sediments and instream aeration, to give satisfied rehabilitation results for shallow lakes. The improvement of the water column conditions, which reflected in increasing of DO content, will promote the P binding in the sediment and prevent the decreases the rate of internal P loading and will affect positively organisms inhabiting the lake (Dondajewska-Pielka et al., 2020). Moreover, the rehabilitation strategies aim not only to restore the water and sediments qualities of the lake but also to maintain the sustainable improvement of the ecosystem.

The lake management strategy, as any environmental management system, is a systematic cycle consisting of four continuous phases, namely, (1) plan, (2) do, (3) check, and (4) act (Aboul Dahab & Shaaban, 2017). Summarizing the cycle, the planning phase is the stage in which the main objectives of the strategy are defined regarding regional, national, and legal obligations and requirements, followed by the do phase, in which the suggested and planned processes and solutions of pollution control are implemented and operated. Then the check phase (or the review) is the performance measurement, which includes continuous monitoring, analyzing the key performance indicators, and evaluating the effectiveness of the applied processes and actions. Finally, the act or improvement phase in which errors and defect points are identified based on the results of the review stage and the appropriate improvement and corrective action(s) are developed (Aboul Dahab & Shaaban, 2017, 2020). Consequently, the current research is considered as a part of the third (check) phase, dealing with a review of the applied lake management strategy of a recently rehabilitated lake. This is achieved by characterizing the performance indicators in the form of measuring the degree of water quality improvements, using various methods such as (i) the scheduled monitoring of indicator parameters of water quality; (ii) comparing the values of those parameters after and before applying any management step; (iii) comparing the water quality parameters with those corresponding values of national and international guidelines; (iv) assessing the suitability of water usage in different purposes (fishing, agricultural, drinking, or recreational….); (v) calculating the water quality index (WQI); (vi) examining the state of eutrophication; and (vii) finally suggesting corrective and improving action(s).

The concept of water quality index (WQI) is commonly applied in the assessment of water quality, and it performs an increasingly significant role in the management of water resources (Hosseini et al., 2021). This concept has been broadly accepted by policymakers and stakeholders as this gives an apparent picture of the water pollution status (Tripathi & Singal, 2019). Moreover, WQI has its specific use for evaluating the suitability of the drinking water supply, irrigation, fish farming, and recreational uses (Fulazzaky, 2009, 2010). The calculation of WQI is based on different physical–chemical and microbiological characteristics. Recently, the principal component analysis (PCA) was used to comprehensively estimate the WQI (Yang et al., 2020).

### Lake Mariut, case study

Regarding Egypt, it is characterized by its northern coastal lake and lagoons (six water bodies), which represent about 25% of the Mediterranean total wetlands. However, these water bodies are the most valuable and are also the vulnerable ecosystem as they receive the wastewater discharged from the watershed (El-Naggar & Rifaat, 2019). Lake Mariut (LM; Mariout, Maryout, Maryut) that is the focus of this research was identified, by the strategic action plan for the Mediterranean, as a hot spot and sensitive area on the northern coast of Egypt, which for several decades has been experiencing a continuous increase in population, development, and environmental degradation. Consequently, it was subjected to a dynamic lake management strategy.

Historically, the LM since the late 60 s of the last century and along 60 years ahead was a victim of uncontrolled urbanization (El Kafrawy et al., 2017; El-Rayis & Abdallah, 2006), and its water quality showed a general downward trend accompanied by a remarkable decline in its importance as a source for the popular Tilapia fish for the Egyptian people and as a place for recreation and nesting wild birds (El Kafrawy & Ahmed, 2020). These deteriorations were reflected in the conversion of LM from the most productive and fertile aquatic habitat in the 1960s, which produced about 60% of the total fish production from the coastal lakes (with a mean annual fish catch > 17,000 tons) to the most polluted and the least productive lake in the 1990s (with a mean annual fish catch of 4000 tons) (Abaza et al., 2009; El-Rayis et al., 1997; Shaaban et al., 2021). Due to this severe water pollution and its consequences, LM attracted the attention of researchers and decision-makers to define the main causes of pollution and adopt some countermeasures to combat the situation (El-Rayis et al., 2019; Shaaban, 2010).

The LM is an example of a continuously rehabilitated lake on the northern Egyptian Coast. It was subjected to several successive rehabilitation steps and corrective actions to improve its water quality and increase the quality and quantity of fish production (Table 1). Based on the literature, it was noticed that the overall lake deterioration was due to the decline of its main basin (MB) ecosystem, where MB acted as a receptor, from 1960, of raw civilian sewage and industrial wastewater discharges. Thus, all the previously implemented steps were obeyed to the preventive approach of lake management and based on decreasing the external nutrient loads from different point sources of pollution. According to those steps, LM during the last century till now has possessed by five successive distinct periods depending on the type, quantity, and quality of the wastewater discharged in its main basin (MB) as summarized in Table 1.

**Fig. 1:**
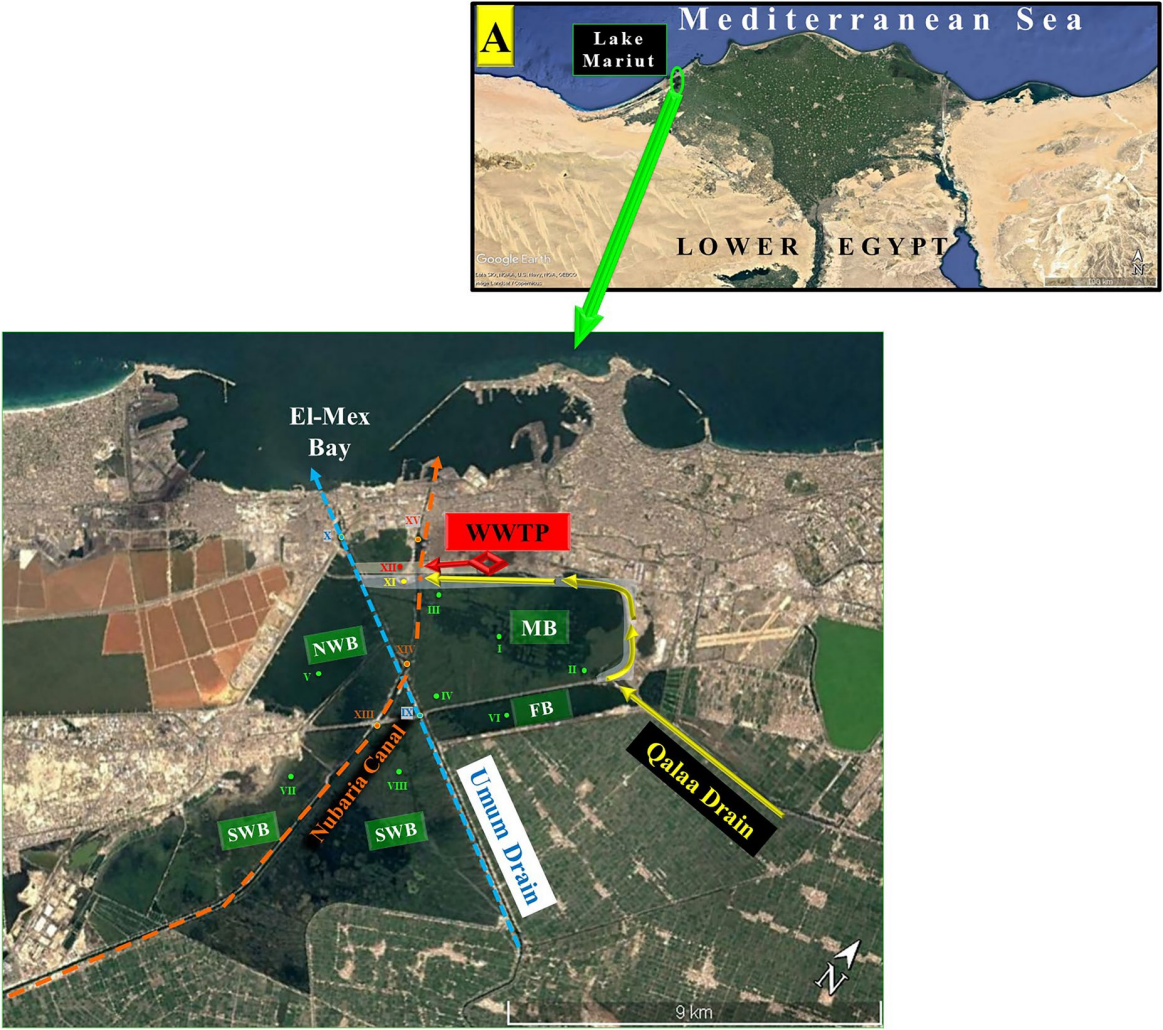
A satellite image of different Lake Mariut Basins (LMBs) and their drainage system showing the location of the sampling stations

Regarding the rehabilitation step, in 1995, all sewage and industrial wastewaters were collected in the constructed east and west wastewater treatment plants (EWTP and WWTP, respectively), and primarily treated before their discharges indirectly and directly to LM via Qalaa Drain (QD; Kallaa, EL-Kallaa, Kalaa, Qalah) and WWTP effluent, respectively (Donia et al., 2016; El-Rayis, 2005; Shreadah et al., 2020) (Table 1). Consequently, in the third period, however, there was a noticeable water quality improvement (for a while); the organic matter and nutrient load discharged were not sufficiently reduced and remained relatively high (Abdallah, 2003). Subsequently, the water quality got worse again, and MB became hypereutrophic, accompanied by a complete depletion in DO and intermittent evasion of toxic hydrogen sulfide (H_2_S) gas, especially in front of two defined hot point land-based sources: one at the southeastern part of MB, where the outlet of the agricultural polluted QD contaminated with the discharge of the EWTP effluent (El-Rayis et al., 2019). The other point source was in front of WWTP mainly industrial discharge at a site laying at the NW side of this basin (MB). Due to the low efficiency of that management step, and as a corrective action, the further rehabilitation step was applied (Table 1). The diversion of the pollution point sources was adopted in 2010 as the cheapest and fastest solution for the problem to alleviate or reduce water pollution. This diversion was attained by discharging directly into the lower reach of the huge agricultural Umum Drain (UD; Omum, Umoum, El-Ommum), before pumping into the sea at El-Mex Bay (SE Mediterranean Sea; Fig. 1). The water quality of MB and its two lately diverted polluted drains was monitored 3 years after the diversion step by Shaaban (2015), during the fourth period (Table 1). The result of the performance indicators of water quality showed that the water of the MB was improved again (to be of the medium condition according to Water Quality Index (WQI)), and became well aerated with DO concentration value of > 10 mg/l and DO saturation > 130% instead of the DO depletion (Shreadah et al., 2020). This improvement was reflected in a remarkable increase in the annual fish production (about 12,301 tons in 2015) and the quality of fish flesh has been improved and become safe for human consumption (Shaaban et al., 2021). However, the MB still showed hypereutrophication conditions as a result of internal TP loading from sediments (Shreadah et al., 2020). Thus, the continuous monitoring of LM water quality is essential not only for reviewing and evaluating the efficiency of the last implemented rehabilitation step but also for examining its sustainability especially under the recent situation which is coincided with lowering the water column depth of LM and applying the secondary treatment process at EWTP. Moreover, the studying of the water quality of the drainage system (diverted drains and feeding drain and canal) is necessary for understanding the surrounding of LM and giving an idea about the availability of these water recourses in solving water sacristy problems. On other hand, however, LM consists of 4 main basins; the MB was the most attracted and studied basin. A few studies were focused on the water quality of the other basins, such as the monitoring periodical program of northern lakes that implemented by the Egyptian Environmental Affairs Agency (EEAA) and the National Institute of Oceanography and Fisheries.

Accordingly, the present study aims to assess the actual water quality situation of LM basins, after 8 years of implementation the diversion of point sources of pollution away from MB of LM, in addition to the drainage system of the LM, as a follow-up monitoring program. In addition, the study targets the evaluation of the water quality of the other basins, and measure if those basins are affected, or not, by the successive steps of the MB rehabilitation and by lowering the water depth of the LM. The study considers this work can be considered as a complementary one, to the previous investigation made by Shreadah et al. (2020) after 3 years of wastewater diversion step, to examine if the adopted rehabilitation program was efficient enough or requires modification(s) and/or suggesting another solution(s) as a corrective action(s). To achieve these objectives, the current spatiotemporal changes of selected water quality parameters and trophic status were examined.

## Material and methods

### Study area

Lake Mariut (LM) is an inland closed shallow lake (with a mean depth of 1 m) that lies south of Alexandria City. It is a depression that extends for about 20 km between 31°01′48″ and 31°10′30″N and 29°49′48″ and 29°57′00″E. Its bottom is below the sea level by value about 3.25 m and separated from the neighboring Mediterranean Sea by a ridge called Abuser (Shaaban et al., 2021). The lake (LM) is artificially subdivided into four principal basins by Desert Road and Umum Drain (UD; Omum, Umoum, El-Ommum). The four basins are, shown in Fig. 1, called Main Basin (MB), Fishery Basin (FB), North-Western Basin (NWB), and South-Western Basin (SWB), with different surface areas of about 24.3, 12.1, 28.3, and 4.1 km^2^, respectively. The last basin (SWB) is split into two subbasins, namely, SWB_2000_ and SWB_5000_, due to crossing of UD through (Fig. 1). As a result, there is a semi-restricted movement of water, fish, and fishermen, causing each basin to function independently and have unique characteristics.

Before 2010, MB was used to be filled with water from three main resources: (1) partly from UD (the huge agricultural drain its course is lining west side of MB and at the same time is lining NWB (at its eastern side) and go through SWBs, (2) Qalaa Drain (QD is also an agricultural one but mixed with the effluent of Alexandria EWTP and was discharged at the southeast side of MB), and (3) the industrial effluent of the WWTP that was pouring directly at NW side of MB. After 2010, as mentioned before, the last two polluted wastewaters have been diverted to pour downstream of UD (El-Rayis et al., 2019; Shreadah et al., 2020). The current discharge rates (at the 5th period, after 2015) of the different water sources related to LM are summarized in Table 1.

### Sampling time and sites

Surface water samples were collected two times, July and November 2018, from 15 selected locations as shown in Fig. 1, representing the different basins of LM and its diverted drains. The details of the name and position of each sampling station location are presented in Table S1 (supplementary materials).

### Methods of analysis

The water quality parameters were assessed in situ. The water temperature was determined via the calibrated graduated thermometer, transparency was detected using the Secchi disk, the hydrogen ion concentration (pH) was measured by the portable pH-meter (Cyberscan^10^pH, pre-calibrated against standard buffer solutions of 4.1, 7.0, and 10.0), and the salinity was determined using portable conductivity meter (CRISON CM-35, Spain, pre-calibrated against conductivity standard reference solutions of 147 µS/cm, 1413 µS/cm, and 12.88 mS/cm).

The concentrations of DO/H_2_S were measured according to the Winkler method (APHA, 1995) using BOD bottles. In which DO was fixed, in situ, using the manganous solution and alkaline azaid solution, then the developed brownish precipitate of the higher valence state of manganic (hydroxide) was acidified by sulfuric acid (50% v/v). After the dissolution of all the precipitates, the libated iodine, equivalent to DO content, was titrated against a pre-standardized thiosulfate solution using starch as an indicator. While in the case of the presence of H_2_S, the developed white precipitate of manganous sulfide was acidified and oxidized by sulfuric acid (50% v/v) and potassium iodate (0.01 N), then the liberated iodine was back titrated against a pre-standardized thiosulfate solution.

For the measurement of dissolved nutrient salts, including nitrate (NO^−^_3_-N), nitrite (NO^−^_2_-N), ammonia (NH^+^_4_-N), reactive phosphate (PO^−3^_4_-P, DIP), and reactive silicates (SiO_4_-Si), water samples were immediately filtered through a 0.45-μm membrane filter and kept frozen until analysis. Concentrations of dissolved nutrient salts were estimated spectrophotometrically, following Strickland and Parsons (1972) methods.

Total-N (TN) and -P (TP) contents were measured according to Valderrama (1981), using unfiltered water samples. All spectrophotometric analyses were done using the Shimadzu UV–Vis spectrophotometer (model UV-1201).

The values of dissolved inorganic nitrogen (DIN) and organic nitrogen (ON) concentrations were calculated using the formulas of [DIN] = [NO^−^_3_-N] + [NO^−^_2_-N] + [NH^+^_4_-N], and [ON] = [TN] − [DIN], respectively.

Regarding, chlorophyll *a* (Chl-*a*) content, it was measured spectrophotometrically according to Strickland and Parsons (1972), by filtrating a certain volume of the water sample, according to the numerical density of phytoplankton, through a 0.45-μm membrane filter. The pigments were extracted overnight using acetone (90%) from the filtered phytoplankton cells. All precautions about the quality control and quality assurance of all studied parameters are considered during sampling, preservation, and analysis.

### Data analysis

The results of water quality parameters were statistically treated by applying the following:

1. The cluster analysis, using the software of STATISTICA 10, organize data into meaningful structure and categories.
2. The principal component analysis (PCA), using IBM SPSS Statistics 25.0, to reduce data and extract a small number of latent factors for analyzing relationships among the observed variables, in addition to easing the evaluation of water quality for each station.
3. The paired sample *t*-test, using IBM SPSS Statistics 25.0, to compare and evaluate the water quality situation of MB after 3 and 8 years of wastewater diversions.

On other hand, the trophic status of LMBs (MB, FB, NWB, and SWB), diverted drains (QD and WWTP), feeding drain (UD), and the canal (NC) were measured using the trophic state criteria (TSC), the trophic state index (TSI), and the nutrient (TN and TP) loading criteria.

## Results and discussion

### Water quality parameters

**Fig. 2:**
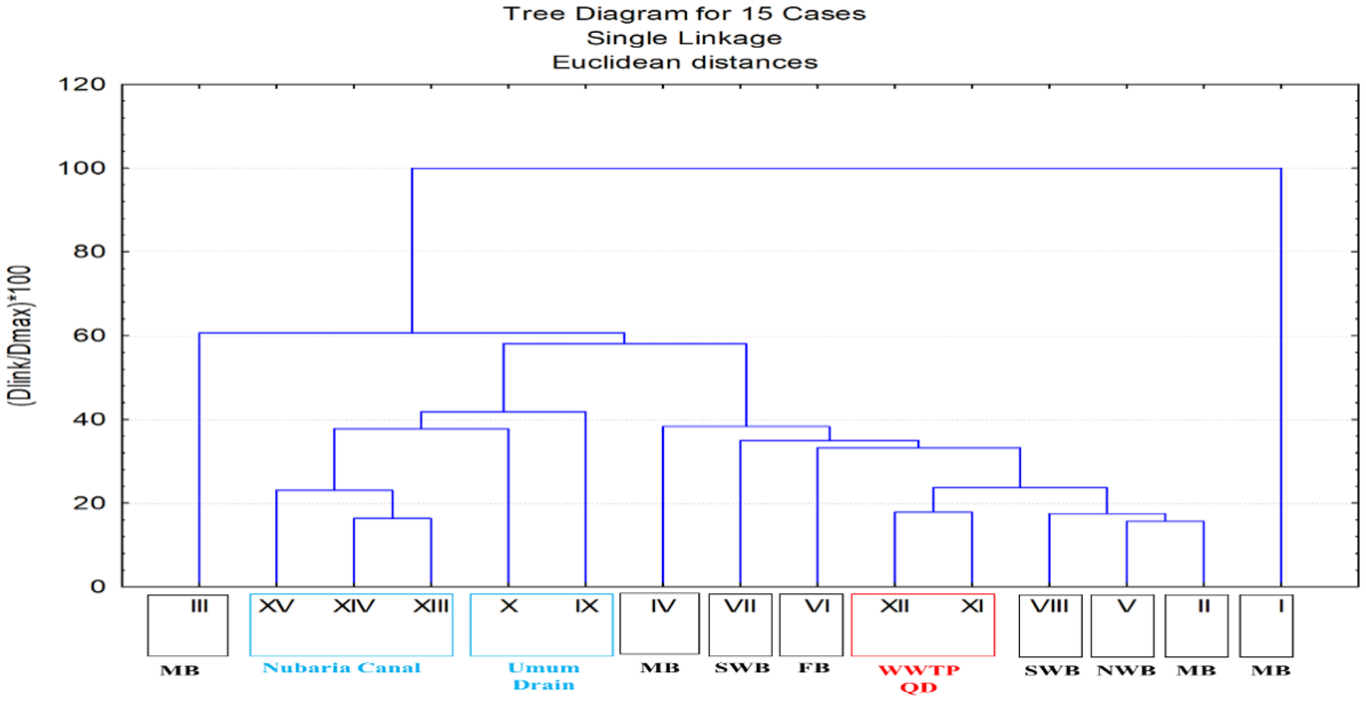
Cluster tree of Lake Mariut Basins (LMBs; Main Basin, MB; Southwest Basin, SWB; Northwest Basin, NWB; and Fishery Basin, FB) and their drainage water system Umum Drain (UD), the diverted Qalaa Drain (QD), West wastewater treatment plants (WWTP) effluent, and Nubaria Canal (NC) during the study period

To represent, classify, and discuss the current situation of different Lake Mariut Basins (LMBs) and their drainage water system (drains and canal), the cluster analysis was employed using the obtained data of water quality parameters and nutrients at the monitoring locations covering the study area. The cluster analysis showed that stations I and III (center and northwest corner of MB, respectively) had their unique characteristics with the dissimilarity of 100 and 60%, respectively, from the study area (Fig. 2). The feeding UD and NC are grouped in one cluster, while LMBs and the diverted drains are to some extent close to each other with a similarity (> 60%). Consequently, the present results can be illustrated in terms of three categories as follow: LMBs, diverted drains, and feeding UD and NC.

**Fig. 3:**
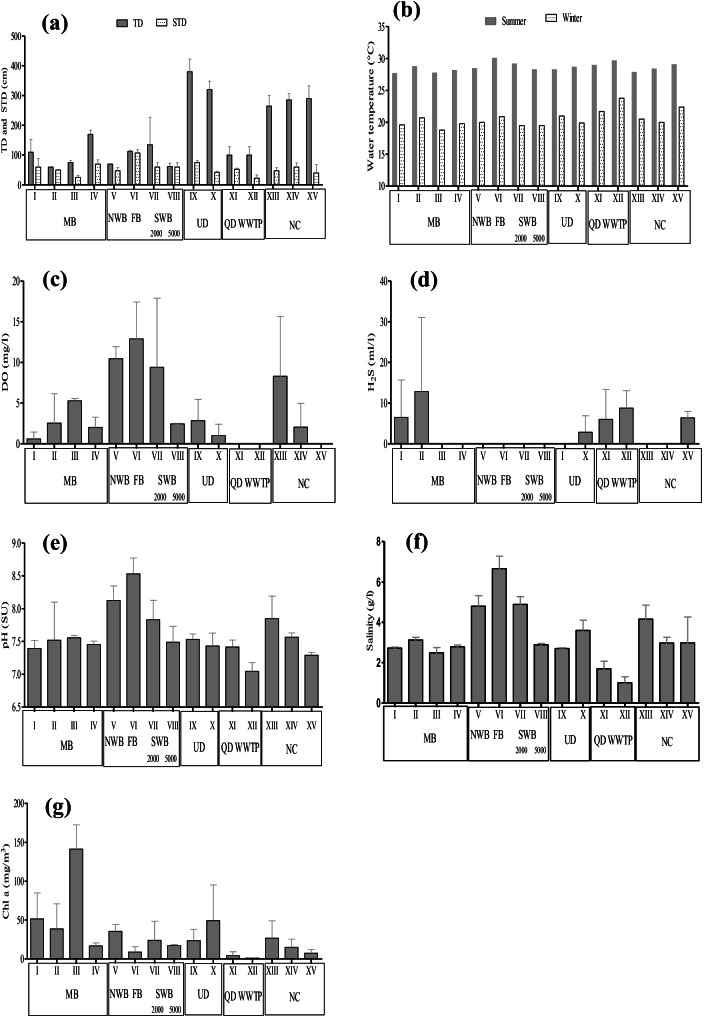
Distribution of **a** total depth (TD) and Secchi disk transparency depth (STD), **b** water temperature, **c** dissolved oxygen (DO), **d** hydrogen sulfide (H_2_S), **e** pH, **f** salinity, and **g** chlorophyll *a*, concentrations in the surface waters of Lake Mariut basins (LMBs), and their drainage water system

LMBs were characterized by their shallowness with an average of 1 m, in addition to the diverted drains from MB (QD and WWTP effluent), while UD and NC were deeper (with an average of about 3-m depth; Fig. 3a). It is worth mentioning that during the present study the surface water in UD was about 1 m lower than that recorded earlier in 2013 (Shreadah et al., 2020), reflecting shortage of the agricultural drainage water by amount reaching about 3 × 10^6^ m^3^/day that could be reused after treatment for plantation of about a half-million acre at west desert (Ministry of the Water Resource and Irrigation, personal communication). This has led some parts of the lake to get dry. This results in agreement with the current monitoring program implemented (EMPNL, 2018, 2020), where about 61% of total area LM have a total depth between 0.5 and 1.0 m.

The obtained results show that the water temperature of LMBs was generally varied between a minimum of 18.8 °C in winter and a maximum of 30.1 °C in summer. This range has coincided with those obtained by EMPNL (2018, 2020) and Farouk et al. (2020), where the water temperatures have fluctuated between 16 and 33 °C. The current results were within the desirable optimum condition for fish culturing (Bhatnagar & Devi, 2013; El Zokm et al., 2018).

Also, the water of LMBs was nearly transparent at both FB and SWB (with STD/TD of > 90%), with STD values of about 110 and 60 cm, respectively. While in MB water the STD/TD % was about 50% less on average, and STD average value of about 50 cm (Fig. 3b). On other hand, the drainage water of the deep UD and NC and each of the polluted shallow QD and WWTP drains were even lower STD/TD of 23 ± 14%, and average STD values of 60, 50, 50, and 25 cm, respectively. The last was in agreement with that recorded by Shreadah et al. (2020), who attributed the low transparency, of the polluted drains, to the dark color observed during sampling, colloidal sulfide metals, and possible loading of the polluted water with dark dissolved and particulate complex organic matter. Moreover, there was a historical agreement that QD is less transparent than that corresponding one of UD. Regarding DO, extreme wide fluctuation in the concentrations over the study area were recorded from nil for the water of the polluted QD, WWTP effluent, and downstream parts of UD and NC, to a high average value of 12.9 ± 4.5 mg O_2_/l (oversaturation, of average % oxygen saturation 169) for the water of FB (Fig. 3c). Therefore, the order of abundance in DO in the study lake was FB (12.9 mg O_2_/l) > NWB (10.5 mg O_2_/l) > SWB (6.0 mg O_2_/l) > MB (2.6 mg O_2_/l). This is in agreement with that mentioned by Abd El-Alkhoris et al. (2020), which recorded the relatively high DO contents in different LMBs (average of 8 mg O_2_/l), in contrast to QD water (complete DO depletion). The standard guideline suitable for aquaculture purposes (Rachman & Adi, 2005) is > 3 mg/l. Therefore, MB water is in a critical stage particularly at the zones of stations I and II (center and the southeast corner of MB, respectively, away from the slightly oxygenated water supply of UD), where H_2_S gas was detected in summer. It seems likely that relatively high temperature accelerates the aerobic decomposition rate of organic matter. Also reflects the existence of remnant oxygen-consuming matter mostly those impeded with the old bottom spoiled organic-rich sediments (El-Rayis et al., 1994). The sluggishness of the water in this basin could be another reason for DO declining. Further the current basin is slightly fed with less oxygenated water at its lonely opening southwest corner from UD, causing limited expansion of oxic water to the middle and eastern parts of MB; only the western part contained DO. Certainly, a solution for such a critical problem is urgently needed to regain the health condition of this important basin and therefore for the fish catch.

There is an observable decline in the current DO content of MB (fifth rehabilitation period) relative to previously recorded data during former periods of lake rehabilitation (Table 2). However, during the fourth rehabilitation period (2010–2015), a pronounced increase in DO concentration was noticed reflecting the effect of insulation of the QD and WWTP (with high organic load, H_2_S-bearing water).

**Table 1:**
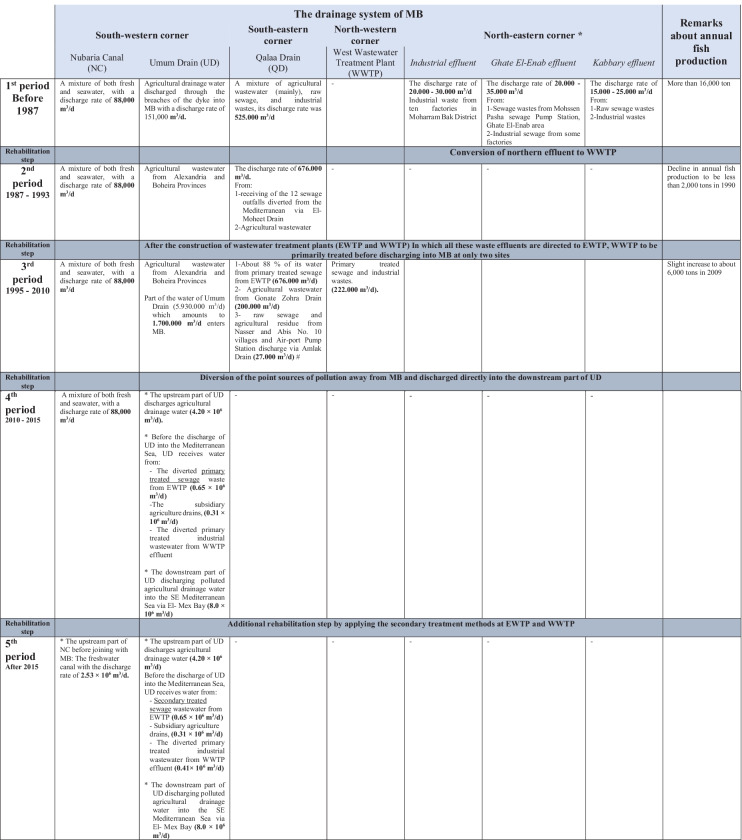
Types and quantities of wastewaters discharged into the main basin (MB) of Lake Mariut from different sources through five successive periods from 1960 to now

# = El-Rayis and Hinckely, 1998, ^*^ = El-Sharkawi, 1991

Focusing on the drainage system, the adverse effect of the high organic matter load is not only restricted on DO depletion and appearance of malodorous H_2_S gas at each of QD and WWTP drain (average 7.4 ml H_2_S/l) but also responsible for converting the oxic water of the proper NC and UD_downstream_ into anoxic one (Fig. 3d). By highlighting the DO content of UD water before joining MB (station IX), in the present work there was a remarkable decline to about one third (2.0 mg O_2_/l equivalent 25% of O_2_ saturation) relative to the previously recorded in 2007 and 2013, where the respective mean values were 6.5 and 8.8 mg O_2_/l (equivalent to 75 and 102% oxygen saturation; Shaaban, 2010; Shreadah et al., 2020, respectively). This drastic decrease often coincided with the latest strategy of the Egyptian Ministry of Water Resources and Irrigation in abstracting about 3 mcm/d from UD, which in turn has led to lowering the water level to − 325 cm (below the sea level) in this drain. This situation consequently has led part of the DO depleted water of SWB_5000_ (station VIII, 2.45 mg DO/l) to drain into the upper reach of UD. These often are the causes of the general decline of DO content noticed in the water of this essential drain.

The pH values fluctuated from 6.95, slightly acidic, in the industrial WWTP to 8.70 at FB (Fig. 3e). The change in the basins was not much and they were in the order: MB (7.48) ≤ SWB_5000_ (7.49) < SWB_2000_ (7.83) < NWB (8.13) < FB (8.53). This distribution is more or less in agreement with that obtained by EMPNL (2018, 2020) and Farouk et al. (2020). The relatively high pH value in FB reflected the role played by the presence of condensed aquatic plants there in the removal of ambient CO_2_ during photosynthetic activities, and thence in raising pH. This observation was previously noticed by El-Rayis et al. (2019). It is worthy to mention that the current pH levels were within the recommended range (5.5–9.5) suggested by the Central Pollution Control Board for the inland waters (CPCB, 1995), and the proposed range (6.0–9.0) by the European Union (EU) for fisheries and aquatic life. By tracking the pH values during different rehabilitation periods (Table 2), a slight decline in the current pH values was noticed from the 4th period (after the diversion of QD and WWTP effluent), to be close to those values recorded just before the diversion (Abdallah, 2003; Shaaban, 2010).

The lower pH values were noticed in QD (7.05); this could be due to the liberation of H_2_S and CO_2_ resulting from anaerobic decomposition processes of organic matter occurring there. It is worthy to mention that QD water always attained slightly acidic and neutral due to its anoxic sewage wastewater, which is verified by an inverse significant relationship between pH and H_2_S (*r* =  − 0.61, *p* < 0.05), especially in high temperatures (summer; July) coincided with increasing the decomposition (oxidation) rate of organic matter.

On other hand, the summer season (July) was exhibited the lowest pH values compared with the winter (November). This could be a result of an increase in water temperature that accelerates the rate of decomposition of organic matter. It is worthy to mention that the distribution pattern pH values at waters of LMBs were previously recorded on the alkali side (Abd El-Alkhoris et al., 2020).

The salinity values of different LMBs were varied between 2.31 and 7.10 g/l and showed the following decreasing order: the FB > NWB ≥ SWB_2000_ > MB = SWB_5000_ (Fig. 3f). It coincided with the previous observation by Farouk et al. (2020), where FB (6.7 ± 0.6 g/l) is a closed basin with a high evaporation rate and no source of direct freshwater discharge and its water source is from Abis PS (with relatively high salinity about 4 g/l; Shaaban, 2010). The horizontal distribution of salinity over the study area confirms the fact that the source of water of both MB (2.78 ± 0.27 g/l) and SWB_5000_ (2.89 ± 0.06 g/l) are from UD (2.70 ± 0.04 g/l) water. Meanwhile, the waters of diverted drains, QD and WWTP, were concurrent with the freshwater salinity (1.35 ± 0.48 g/l) reflecting the role of municipal sewage water (> 88%) in decreasing the salinity of the drain water. In contrast the water of navigational canal (NC) and the agricultural drain (UD) attained a relatively high salinity (3.30 ± 0.67 g/l).

The fluctuation in salinity values between basins was also found by the monitoring program (EMPNL, 2018, 2020). For MB, however, the average salinity (or the calculated chlorosity) of the current study did not show changes when compared with those values recorded after the diversion of pollution sources (fourth period); it is higher than those values of the third period (the period between the constructions of EWTP and WWTP and before the pollution source diversion, 1995–2010) (Table 2).

The Chl-*a*, the indicator of phytoplankton abundance and biomass (Ahmed, 2020), is of values ranged between higher levels in the waters of a relatively high residence time of LMBs (average 43 mg/m^3^) to lower Chl-*a* level (< 8 mg/m^3^) of anoxic QD and WWTP drain. This notice agrees with that observed by Abd El-Alkhoris et al. (2020). The low Chl-*a* content in QD and WWTP drain reflects unfavorable conditions for algal plants due to evasion of toxic H_2_S. The order of Chl-*a* abundance between LM basins is MB > NWB > LMFB = SWB (Fig. 3g). Generally, the maximum Chl-*a* content was recorded in the summer season, coinciding with low transparency and high DO concentration, reflecting the role of photosynthetic activities. Focusing on MB, however, there was an observable decrease in Chl-*a* content to about one third of that value observed in 2013 (4th rehabilitation period); it is relatively close to those values recorded in 1995 (3rd rehabilitation period) as illustrated in Table 2.

Monitoring of nutrient (N and P) levels has a significant response on the water quality and food webs at the lake (Somura et al., 2018). And the nutrient content determines the amount of phytoplankton and algal biomass, reflected in eutrophication status and related to total fish production (El Zokm et al., 2018).

**Fig. 4:**
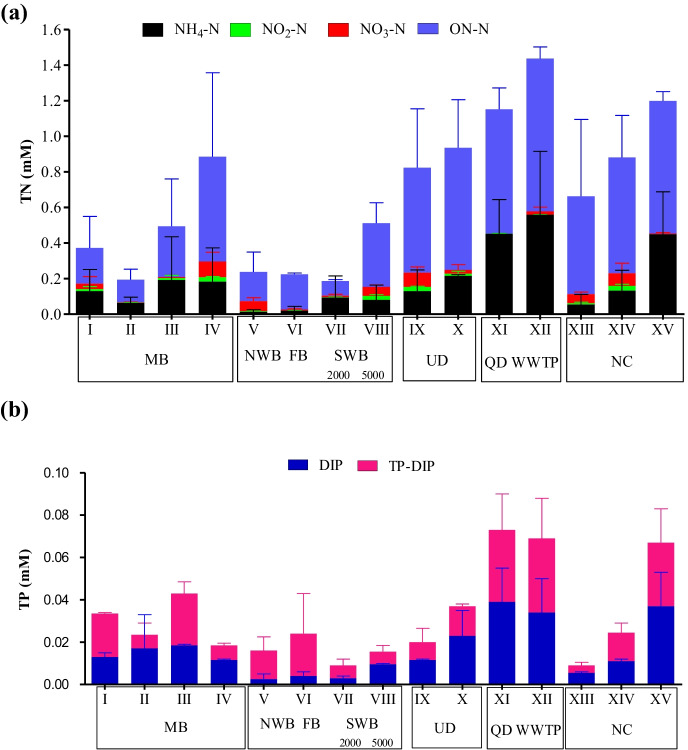
Distribution of **a** total nitrogen (TN) and **b** total phosphorus (TP) together with their different species (mM) in the surface waters of Lake Mariut Basins (LMBs) and their drainage water system

In the current study (Fig. 4a), the TN content (with an average value > 1 mM) of the drainage water system (drains and canal) sustained at least 3 times higher than those recorded at different LMBs (with an average value < 0.4 mM). Moreover, most of the TN was in ON form in the study area, particularly, in the warm season (July). The order of TN abundance was SWB_5000_ (7.2 mg/l, 0.51 mM) > MB (6.8 mg/l, 0.49 mM) > NWB (3.3 mg/l, 0.24 mM) ≥ FB (3.1 mg/l, 0.22 mM) > SWB_2000_ (2.6 mg/l, 0.19 mM), which reflect the declining in the TN content regarding to previous studies (Abd El-Alkhoris et al., 2020) by at least 25%.

The distribution pattern of DIN levels revealed that the least DIN values were at FB (0.02 mM) followed by NWB (0.07 mM), SWB (0.13 mM), and MB (the relatively high DIN content with an average of 0.19 mM). Moreover, July (hot season) attained the lowest values of DIN (< 0.1 mM) compared with relatively higher ones in November (cold season). Most anoxic waters of the diverted drains (QD and WWTP), downstream waters of UD and NC, were characterized by the elevated DIN content relative to aerated stagnant waters of LMBs. On other hand, the downstream of the running water of both UD and NC were, always, exhibited an elevated DIN content than their upstream waters.

The NH_4_ has constituted a considerable part of DIN, particularly in the anoxic waters (> 90%) as illustrated in Fig. 4a, revealing the reducing conditions. This was an indication of the ammonification and/or deamination processes, which were concurrent with a decline in the NO_3_^−^ concentration.

By comparing the N-form concentration values of the current study (8 years after the diversion of pollution sources) with the corresponding earlier values (Table 3), it was noticed that (1) NH_4_ re-increased again after its recorded depletion (3 years after the diversion), but still lower than those values obtained in the 3rd period of rehabilitation); (2) DIN was showed a relatively same trend of NH_4_; and (3) generally, there was a decreasing trend in TN content from the 3rd rehabilitation period till current period.

**Table 2:** A comparison between the level of the present values of water quality parameters in the surface waters of Lake Mariut Main Basin (MB) with those previously reported in the same locations

Rehabilitation period	Sampling date	STD (cm)	DO (mg/l)	(% DO sat.)	H_2_S (mg S/l)	pH (SU)	Clv (g/l)	Chl-*a* (mg/m^3^)	Reference
**I**	2018	20–80 **(49)**	ND–5.5 **(2.62)**	**(31.1)**	ND–25.7 **(4.8)**	7.11–7.93 **(7.48)**	1.11–1.55^**^ (1.34)^**^	7.9–163.4 **(59.6)**	Present study
**II**	2013	10–110 **(55)**	ND–31.2 (**14.3)**	0–311 **(151)**	ND–57.7 **(43.1)**	7.26–8.68 **(7.83)**	1.01–1.99 **(1.34)**	15.9–419.9 (**174.0)**	Shreadah et al. (2020)
**III**	2006–2007	30–55 **(43)**^**w**^	3.7–9.3 **(5.8)**^**w**^	47–98 **(66)**^**W**^	-	7.21–7.51 **(7.36)**^**W**^	0.82–1.26 **(1.01)**^**W**^	3.2–31.3 (**10.2)**^**W**^	Shaaban (2010)
		15–20 **(19)**^**E**^	-	**-**	10.0–104.9 **(37.6)**^**E**^	6.76–7.20 **(7.02)**^**E**^	0.47–0.71 **(0.60)**^**E**^	3.8–73.4 **(26.4)**^**E**^	
	1998–1999	-	1.5–12.0 (**6.2)**	**-**	0.1–6.7 **(2.4)**	7.0–8.6 **(7.71)**	1.30–3.20 **(1.92)**	-	Khalil et al. (2000)
	1995–1996	10–80 **(34)**	ND–13.7 (**2.5)**	**(24.7)**	ND–40.3 **(6.9)**	6.3–9.1 **(7.60)**	0.60–1.66 **(1.03)**	8.0–154.5 (**49.5)**	Abdallah (2003)
		30–90 **(60)**	ND–16.1 (**5.6)**	**-**	ND–12.0 **(1)**	7.3–8.0 **(7.70)**	0.24–4.26 **(1.29)**	0.1–484.0 (**70.6)**	Abdel Aziz and Aboul Ezz (2004)
		-	-	**-**	0.1–12.0	-	0.89–1.40	-	El-Rayis et al. (1998)
		-	-	**-**	-	6.8–8.5	-	-	El-Rayis and Hinckely (1998)
		-	ND–11.0 (**3.4)**	**-**	-	-	-	0.1–390.0 (**67.8)**	Essa and Faltas (1997)
**IV**	1982	10–90 **(34)**	ND–21.9	**-**	-	7.1–11.0	-	-	Abdalla et al. (1991)
	1980–1981	-	-	-	-	(**7.56)**	(**1.42)**	-	Saad (1983)
	1975–1977	-	ND–16.6	-	0.0–78.9	-	-	-	Samaan et al. (1988)
		-	-	-	-	7.0–9.6	0.27–1.76 **(1.63)**	-	Abdel-Moniem et al. (1987)
		0.1	0.2–11.0	-	-	-	-	-	El-Sharkawy (1978)
	1968–1969		-	-	-	7.35–9.50 **(8.31)**	1.38–2.24	-	Samaan and Abdallah (1981)
	1957–1958		2.2–18.1	25–191	-	8.1–9.5	1.52–6.44	-	Wahby (1961)

The bold value between parentheses refers to the average value. The Capital letters E and W refer to the Eastern and Western parts of MB.* ND* refers to not detected

*I* 5th period (after 2015), *II* 4th period (2010–2015), *III* 3rd period (1995–2010), *IV* 1st period (before 1987)

**Calculated chlorosity from the relation betweenClv and salinity from formula obtained by Shaaban (2010), Clv (g/l) = salinity (g/l)/2.0747

Like TN, the current TP concentrations of LMBs were generally lower (by about three times of magnitude) compared with those of the drainage water system (Fig. 4b). Regarding LMBs, the order of abundance of TP was MB (0.92 mg/l, 0.030 mM) > FB (0.75 mg/l, 0.024 mM) > NWB = SWB_5000_ (0.49 mg/l, 0.016 mM) > SWB_2000_ (0.29 mg/l, 0.009 mM). However, MB showed a relative decrease in TP levels when compared with the previous studies (Abd El-Alkhoris et al., 2020); this situation was reversed for the other basins, where the TP contents of the present study were higher than the previous studies.

The extremely elevated levels of TP (> 0.07 mM) were observed at the diverted polluted drains QD and WWTP, beside the downstream part of UD (Fig. 4b). The high TP levels in the diverted drains are due to the massive amount of sewage discharge usually loaded with P.

The percentage of DIP to TP was higher in summer (July) relative to that in winter (November); this could be attributed to the increase of domestic discharge that was enriched with polyphosphates (in commercial soaps and detergents), which are quickly hydrolyzed and yielded to the production of orthophosphate species in aqueous solution.

Comparing the present study results with the guidelines of the Egyptian Ministry of Water Resources and Irrigation “Law 48/1982,” for protection of the Nile River and waterways from pollution, i.e., Decree No. 49 in the amended executive regulations of the law by Minister Decision No. 92/2013 (Table 4) revealed that MB and SWB showed elevated levels of most nutrient salts (NH_4_^+^, ON, TN, and TP) than those recommended by Egyptian Environmental Affairs Agency (EEAA); however, average DO concentrations of MB were lower than permissible limits. The pH values were within the recommended range. On the other hand, the other two lake basin waters of NWB and FB were within the allowable boundaries for most parameters excluding ON content.

**Table 3:** A comparison between the range and (average) levels of the present values of nutrients (μM/l) in the surface waters of Lake Mariut Main Basin (MB) with those previously reported in the same locations

Rehabilitation period	Sampling date	NH_4_-N	NO_2_-N	NO_3_-N	DIN	TN	DIP	TP	Reference
**I**	2018	20–364 **(141)**	0.2–28.1 **(11.7)**	ND–126 **(32.1)**	20–397 **(185)**	117–1119 **(486)**	0.8–33.0 **(14.9)**	2.2–49.1 **(29.7)**	Present study^**^
**II**	2013	ND–354 **(70)**	0.1–52.9 **(16.2)**	1–67 (**31)**	43–363 **(117)**	177–859 **(607)**	10.5–34.6 **(16.3)**	19.9–93.8 **(41.4)**	Shreadah et al. (2020)
**III**	2006–2007	ND–138 **(46)**^**w**^	6.1–42.3 **(19.8)**^**W**^	28–166 (**72)**^**W**^	71–224 **(138)**^**W**^	245–1176 **(488)**^**W**^	1.9–10.8 **(6.7)**^**W**^	6.5–21.6 **(12.8)**^**w**^	Shaaban (2010)
		354–858 **(502)**^**E**^	0.1–2.2 **(1.1)**^**E**^	ND^E^	356–859 **(503)**^**E**^	674–2683 **(1651)**^**E**^	21.5–90.6 **(63.1)**^**E**^	74.0–1876.9 **(353.8)**^**E**^	
	1998–1999	**(67)**	ND–92. (**18.6)**	0.0–164 **(33)**	-	-	0.7–25.9 **(10.7)**	-	Khalil et al. (2000)
	1995–1996	ND–2206 **(857)**	N.D–37.8 **(6.4)**	N.D–240 **(72)**	**(935)**	1207–6561	4.6–116.9 **(71.5)**	11.4–213.1 **(113)**	Abdallah (2003)
		2–2363 **(579)**	0.1–164.2 **(7.9)**	1–236 **(25)**	**-**	**-**	0.1–218.9 **(72)**	-	Abdel Aziz and Aboul Ezz (2004)
		**(557)**	**(7.9)**	**(24)**			0.3–106.5	-	El-Rayis et al. (1998)
		1.0–2356 **(557)**	**-**	-	**-**	**-**	-	0.1–220.9 **(69.5)**	Essa and Faltas (1997)
**IV**	1982	1–117 **(28)**	-	-	**-**	**-**	3.6–100.1 **(43.3)**	-	Abdalla et al. (1991)
	1975–1977	1–893	**-**	-	**-**	**-**	0.0–623.2	-	Samaan et al. (1988)
		-	-	**(23)**	**-**	**-**	0.0–477.9	-	El-Sharkawy (1978)
	1957–1958	-	ND–6.5	ND–18	**-**	**-**	0.0–249.9	-	Wahby (1961)

*I* 5th period (after 2015), *II* 4th period (2010–2015), *III* 3rd period (1995–2010), *IV* 1st period (before 1987)

### Trophic status

The trophic state can be used for evaluating the efficiency of the restoration program, and for lake categorization. This can be accomplished by applying three definite approaches, namely, (1) trophic state criteria (TSC); (2) trophic state index (TSI); and finally, the nutrient (TN and TP) loading criteria (Premazzi & Chiaudani, 1992).

Based upon the TSC (first approach), some proposed parameters were used for evaluating changes in the ecological conditions of lakes and for qualitative description of the trophic status. The TSC includes abiotic, STD, DO, TP, TN, organic- and inorganic-N, and biotic parameters such as Chl-*a* (Premazzi & Chiaudani, 1992; Thomas et al., 1996; Wetzel, 2001).

**Fig. 5:**
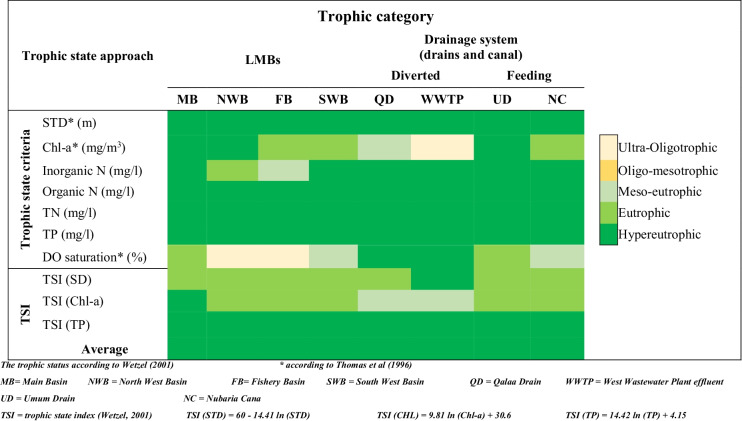
A heat map shows the classification of the study area water according to different trophic status criteria and trophic state index (TSI)

The trophic statuses of the studied lake waters relative to standards were presented in Fig. 5. It reveals that although waters of both NWB and FB were supersaturated with DO and contained relatively low concentrations of inorganic-N forms, their waters were identified as hypereutrophic relative to other standard parameters. While MB water was categorized as hypereutrophic regarding suggested parameters. Additionally, the diverted drain and effluent of QD and WWTP were recognized as dystrophic, with poly-humus, refractory dissolved organic, and brown water which decrease the attenuation of light, causing the decline in Chl-*a* concentration (Carpenter & Pace, 1997).

The calculated TSI using these formulas developed by Carlson (1977) that are based on STD, TP, and Chl-*a* are presented in Fig. 5. The results indicated that the TSIs, according to STD and Chl-*a*, most of the study areas, were grouped as eutrophic waters. On contrary, the TSIs (TP) were classified, the study area, as hypereutrophic. Moreover, the order of TSI values: TSI (TP) > TSI (Chl-*a*) = TSI (STD), proposed that zooplankton grazing or/and N-content are growth-limiting factors of algal biomass production (Brown & Simpson, 2001).

The nutrient loading criteria according to the Vollenweider model (Vollenweider, 1968, 1976) were applied to compare the present study TN and TP loading levels of UD at its upstream and downstream (UD_us_ and UD_ds_,) respectively, with those corresponding permissible and dangerous loading levels () that required to maintain lake in a steady state as a function of its mean depth (Table 5). The results revealed that UD_us_ (the feeding water source of MB after the diversion of QD and WWTP effluent) loads TP lower than the permissible and dangerous loading levels to MB. However, TN loading was exceeding the permissible levels; it was lower than the dangerous loading values. Moreover, levels of TN and TP entering Mex Bay through UD_ds_ were slightly higher than the permissible limit and lower than dangerous loading.

**Table 4:** A comparison between the present study data of MB and its drains with those recommended according to Egyptian Law (92/2013)

Parameters	Unit	Egyptian Law (92/2013)				LMBs				Drainage system (drains and canal)			
		**FW**	**Agri. D**	**TWW**						**Diverted**		**Feeding**	
				**Domestic**	**Industrial**	**MB**	**NWB**	**FB**	**SWB**	**QD**	**WWTP**	**UD**	**NC**
**DO**	mg/l	> 6	> 5	> 4	> 4	**2.6**	10.5	12.9	6.0	**ND**	**ND**	**1.9**	**3.4**
**pH**	SU	6.5–8.5	6.5–8.5	6.0–9.0	6.0–9.0	7.5	8.1	8.5	7.7	7.4	7.1	7.5	7.6
**ON**	mg/l	< 1	-	-	-	**4.2**	**2.3**	**2.8**	**3.1**	9.8	12.0	8.9	9.1
**NH**_**3**_	mg/l	< 0.5	-	-	-	**2.0**	0.2	0.3	**1.2**	6.3	7.8	2.4	3.0
**NO**_**3**_	mg/l	< 2	-	-	-	0.5	0.8	0.1	0.4	ND	0.3	0.7	0.6
**TN**	mg/l	< 3.5	15	-	-	**6.8**	3.3	3.1	**4.9**	16.1	20.1	12.3	12.8
**TP**	mg/l	< 0.5	3	-	-	**0.9**	0.5	**0.8**	0.4	2.3	2.1	0.9	1.0

Bold numbers represent the higher values than recommended guidelines

*FW* guideline for fresh waters subjected to industrial wastewater discharge, *Agri. D* guideline for agricultural drains, *ND* not detected,*-* not mentioned criterion, *TWW* treated wastewater before discharge into non-freshwater aquatic environment, *MB* Main Basin, *NWB* North West Basin, *FB* Fishery Basin, *SWB* South West Basin, *QD* Qalaa Drain, *WWTP* West Wastewater Plant effluent, *UD* Umum Drain, *NC* Nubaria Canal

### Principal Component Analysis (PCA) and Water Quality Index (WQI)

The principal component analysis (PCA), the multivariate method, has been applied on 16 variables (measured parameters) and 15 sampling stations for the two seasons. The PCA was applied to define the parameters responsible for the main variability in water quality variance. The applicability of PCA was verified by the Kaiser–Meyer–Olkin (KMO) and Barlett tests. The computed result values for KMO and Barlett tests were 0.60 (i.e., > 0.5) and 0.00 (i.e., < 0.05), indicating the suitability of the sample and the independence of each variable, respectively. Moreover, the defining of the PC numbers is based on three principles: (1) the eigenvalue (*λ*) of the PC should be more than 1; (2) the percentage of the cumulative variance of PCs is greater than 80%; and (3) the number of PCs should be regulated by the mutation of eigenvalue (Zhang et al., 2019).

The results of the PCA, including loading for the rotated component matrix, eigenvalues for different components, percent, and cumulative percent of variance explained by each component, are shown in Table 6. Based on the outputs, the 16 measured parameters can be replaced by five main PCs explaining about 86.3% of the total variability explaining 24.58%, 21.29%, 16.91%, 12.32%, and 10.23% for PC1 (pH factor), PC2 (NH_4_^+^-N factor), PC3 (NO_2_^−^-N factor), PC4 (total depth, TD factor), and PC5 (Chl-*a* factor), respectively (Table 6). The loading of the variables on the five PCs showed that pH, salinity, DO, and DIP are the dominant variables on PC1 (− 0.90, − 0.84, − 0.80, and 0.73, respectively). The N-forms were the core variables controlling 2nd and 3rd components, where NH_4_ (0.94), DIN (0.93), and TN (0.68) on PC2 and NO_2_ (− 0.91) and NO_3_ (− 0.74) on the PC3. Finally, the TD (0.87) and percentage of STD to TD (− 0.84), in addition to STD (0.75) and Chl-*a* (− 0.87), were the dominant variables on PC4 and PC5, respectively. However, some of these loadings may simply be the results of loadings on other components. For example, chlorophyll loads on component 5 but the drivers of algal biomass (N, P) have already loaded on components 1 and 2. Moreover, the most dominant variables of PC1 were negatively correlated with components and positively correlated to each other. Likely, high significant linear relationship between DO and pH at the meantime the inverse relationship between H_2_S with both DO and pH indicated the presence of liberated H_2_S and CO_2_ weak acids resulted from an occurrence of oxidation (decomposition) processes of organic matter and exhaustion of DO (El-Rayis et al., 2019).

**Table 5:** Values of the permissible loading levels for TN and TP in g/m^2^ yr as well as those values of Umum Drain upstream and downstream (UD_us_ and UD_ds_, respectively) in the present study

**Mean depth**	Wetzel (2001**)**				**Present study**			
	**Permissible loading**		**Dangerous loading**		**MB from UD**_**us**_		**Mex Bay from UD**_**ds**_	
	**N**	**P**	**N**	**P**	**N**	**P**	**N**	**P**
** < 5 m**	< 1.0	< 0.07	> 2.0	> 0.13	1.1	0.06	-	
**10 m**	< 1.5	< 0.10	> 3.0	> 0.20	-		1.6	0.15

On other hand, the PCA has been proven as an efficient and computationally elegant technique for the classification of water samples (Mahapatra et al., 2012; Yang et al., 2020), and for obtaining the water quality index (WQI). The principal component scores were determined with the contribution rate of variance as the weight; then, the composite (comprehensive) score, *F*, was calculated from the equation (Zhang et al., 2019):

F = $\frac{{\lambda }_{1}}{{\lambda }_{1}+{\lambda }_{2}+\dots +{\lambda }_{n}}{F}_{1}+\frac{{\lambda }_{2}}{{\lambda }_{1}+{\lambda }_{2}+\cdots {\lambda }_{n}}{F}_{2}+\dots -\frac{{\lambda }_{n}}{{\dot{\lambda }}_{1}+{\lambda }_{2}+\cdots {\lambda }_{n}}{F}_{n}$where *λ*_*n*_ value corresponds to the variance of the principal component (*n*) and *F*_*n*_ is the calculated factor of the principal component (*n*).

**Fig. 6:**
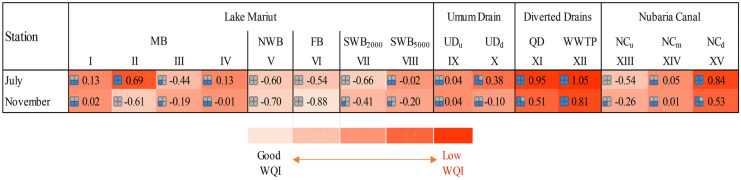
Heat map shows the distribution of the water quality index based on comprehensive score of principal components (*F*) for the surface waters of Lake Mariut Basins (LMBs) and their drainage water system

The results of the present study are illustrated in Fig. 6. The positive and negative values of comprehensive scores of PCs (*F*) do not represent the absolute water quality but represent its relative quality (An et al., 2015). And the (*F*) value is inversely proportional to the water quality; the greater the score value, the more serious the pollution and low-water quality that need corrective action. Accordingly, the water quality of the diverted drain and effluent of QD and WWTP, in addition to the downstream part of NC, was the worst, comprehensive score > 0.5 (Fig. 6). The FB, NWB, and SWB_2000_ showed relatively good water quality when compared with MB (Fig. 6).

### Comparative evaluation of water quality of MB

To evaluate the efficiency of wastewater effluent diversion process on the water quality of MB with time (as MB-rehabilitation step), it can be employed by comparing the current state (present study, 2018) with the older ones in 2013 (Shaaban, 2015; Shreadah et al., 2020), with 5-year interval. It is worthy to mention that sampling stations in 2013 and 2018 were at the same positions and from the same seasons. The average concentration values of selected parameters during 2013 and 2018 are presented in Tables 7 and S2 (supplementary materials).

**Table 6:** Varimax rotated component matrix of the studied parameters in Lake Mariut Basins and their drainage system

Rotated component matrix^a^ 2018–2019					
	Component (*r* = 0.46, *p* > 0.01)				
	1	2	3	4	5
Total depth (TD)	− 0.01	0.04	− 0.22	**0.87**	0.10
Secchi transparency depth (STD)	− 0.31	− 0.32	− 0.21	− 0.15	**0.75**
STD/TD	− 0.17	− 0.22	− 0.02	** − 0.84**	0.37
Water temperature	0.27	** − 0.52**	**0.57**	0.41	0.09
pH	** − 0.90**	− 0.24	0.02	− 0.16	0.12
Salinity	** − 0.84**	− 0.30	0.11	− 0.10	0.21
Dissolved oxygen (DO)	** − 0.80**	− 0.39	0.20	0.03	0.00
Hydrogen sulfide (H_2_S)	**0.67**	− 0.11	**0.55**	− 0.09	0.07
Chlorophyll *a* (Chl-*a*)	− 0.06	− 0.29	0.05	− 0.02	** − 0.87**
Ammonia (NH_4_^+^-N)	0.28	**0.94**	0.16	0.04	− 0.02
Nitrite (NO_2_^−^-N)	0.12	0.03	** − 0.91**	0.10	0.12
Nitrate (NO_3_^−^-N)	0.04	− 0.24	** − 0.74**	0.33	0.23
Dissolved inorganic nitrogen (DIN-N)	0.30	**0.93**	− 0.03	0.10	0.03
Total nitrogen (TN-N)	0.36	**0.68**	0.00	**0.49**	0.12
Dissolved inorganic phosphorus (DIP-P)	**0.73**	0.15	**0.50**	0.20	0.07
Total phosphorus (TP-P)	**0.51**	**0.54**	**0.54**	0.10	0.01
Eigenvalues	6.088	2.839	1.955	1.559	1.372
Variance %	24.58	21.29	16.91	13.32	10.23
Cumulative %	24.58	45.86	62.78	76.10	86.32

Bold values reflected the significant value

Extraction method: principal component analysis. Rotation method: varimax with Kaiser normalization

^a^Rotation converged in 18 iterations

**Table 7:** Results of the paired *t*-test of differences of the environmental and nutrients between the two successive periods after 3 and 8 years of insulation of wastewater effluents away from MB

Paired sample statistics						Paired differences		*t*	Sig. (2-tailed)	Percentage of loss ( −) and gain ( +) in 2018 from 2013
**Pairs (parameters)**	**Year**	***N***	**Mean**	**Standard deviation**	**Mean**		**Standard deviation**			
**TD**	2018	18	176	116	− 42 (− 2.4 to − 81.0		79	− 2.24	0.04	− 19
	2013	18	217	165						
**DO**	2018	18	1.76	2.38	− 8.95 (− 2.3 to − 15.6)		13.44	− 2.82	0.01	− 84
	2013	18	10.70	13.90						
**H**_**2**_**S**	2018	18	4.11	7.10	− 26.92 (− 5.1 to − 48.8)		43.91	− 2.60	0.02	− 87
	2013	18	31.03	46.88						
**TN**	2018	18	0.773	0.444	− 0.60 (− 0.01 to − 1.1)		1.04	− 2.46	0.02	− 44
	2013	18	1.376	1.283						

The results showed, however, that the observable decreasing trend in the concentrations of H_2_S, TN, NO_2_, DIP, and TP by approximately 87, 44, 40, 25, and 20%, respectively, could be an improvement indicator. Those were combined with decreasing in DO concentration (by 84%), and a moderate decline in TD, transparency, and pH by 19, 11, and 2%, respectively, and a slight increase in water salinity by 2%.

Moreover, the previous notice was statistically confirmed by applying the paired *t*-test using the data of the environmental and nutrient parameters between the two successive periods after 3 years (Shaaban, 2015; Shreadah et al., 2020) and 8 years (present study) of diversion. The results revealed significant temporal variations in terms of total depth, DO, H_2_S, and TN (*p* < 0.05), while the other parameters did not show significant differences (Tables 7 and S2, supplementary materials).

Unfortunately, the current results reveal that the subsequent improvement of MB water quality was temporary and fell short of expectations. The re-deterioration was fasted by lowering the water level of UD, the main LM-water source, with its negative impact not only on LMBs but also on the quality of the drain itself.

Accordingly, the implementation of further rehabilitation steps is highly recommended. Strengthening the east bank of UD separating it from MB is a must to keep the water surface level constant, dredging the spoiled organic-rich sediments to lower any internal load of nutrients and deepening the basin too. If the wastewater of the EWTP (about half mcm/d) becomes now secondary treated it is better to allow it to flow again directly into MB. The excessive water is therefore allowed to flow over the bank into UD and not the reverse. This will replenish water loss by evaporation and/or evapotranspiration and seepage from this basin besides overcoming elevation in its water salinity.

## Conclusion

Egypt has made significant efforts to mitigate the pollution of LM over the past 6 decades, but the efficiency of these restoration activities remains unstable; accordingly, there were 5 distinct successive rehabilitation periods (from before 1987 till now). The present study describes and evaluates the Lake Mariut (LM) basins and drainage water system state after 8 years of implementing the last distinct restoration step (the prevention of pollution point sources) of this formerly heavily polluted lake.

Although the major standards of the remediation and restoration of Lake Main Basin (MB) are well characterized, the aftereffects of the implemented measure can be disappointing after a relatively short period (around 8 years). The deterioration in most water quality parameters was observed, again, which is often attributed to the continuous internal fertilization (nutrient loading) from nutrient-rich sediment. Thus, the application of more efficient and applicable solution(s) is A MUST. The suggested solutions recommended are summarized as follows:

1. Mounting the MB, particularly the UD east bank, to make the elevation of the surface water inside MB independent of that in the drain itself is an urgent request. The same is also needed for the other basins.
2. Dredging the spoiled nutrient and organic-rich sediments and deep-rooted plants.
3. Allow the EWTP effluent, which is now secondary treated (i.e., it becomes of low and acceptable organic matter and nutrient contents) to pour again directly into MB instead of discarding into the sea via UD_downstream_
4. 1- Integrated management of the drainage system and positioning the place of drain’s entrance (feeding water source) is recommended.

Of course, it is better to adopt the three suggestions together and at once or in a sequent two main steps: one includes both suggestion nos. 1 and 2 simultaneously, followed by the last one no. 3.

Lastly, it would be advisable to implement the previous recommendations accompanied by frequent continuous monitoring of water quality parameters including physical, chemical, and biological (fauna and flora) characteristics for LMBs, diverted drains (QD), effluent (WWTP), and feeding drain (UD) and the canal (NC).

## Supplementary information

Below is the link to the electronic supplementary material.

Supplementary file1 (DOCX 19 KB)

## Data Availability

Some of the data generated or analyzed during this study are included in this published article. The other datasets used and/or analyzed during the current study are available from the author on reasonable request.
