# Supplementary material for: Water quality and trophic status of Lake Mariut in Egypt and its drainage water after 8-year diversion
Source: Environ Monit Assess. 2022 Apr 28;194(6):392. doi: 10.1007/s10661-022-10009-8 (PMC9046344; doi:10.1007/s10661-022-10009-8)
Supplement: Supplementary file 1 — Supplementary file1 (DOCX 19 KB) [file 10661_2022_10009_MOESM1_ESM.docx]

**Appendix A**

**Table (S1)– Name and position of each sampling station location**

| Area | | station No. | Location description |
| --- | --- | --- | --- |
| Lake Mariut  (LM) | MB | I  II  III  IV | Centre of MB (6000 feddan)  Southeast corner of MB opposite to the entrance of QD inflow at Alexandria-Cairo Desert Road  Northwest corner of MB neighbouring UD  Southwest corner of MB neighbouring UD |
|  | NWB | V | Centre of NWB (3000 feddan) |
|  | FB | VI | Centre of FB (1000 feddan) |
|  | SWB | VII  VIII | West of SWB (2000 feddan)  East of SWB (5000 feddan) |
| Umum Drain  (UD) | | IX  X | Upstream of UD at Alexandria-Cairo Desert Road Downstream of UD prior Mex Bay |
| Diverted Drains | QD  WWTP | XI  XII | Downstream of QD before joining with UD  In front of WWTP discharge |
| Nubaria Canal  (NC) | | XIII  XIV  XV | Upstream of NC at Desert Road  Mixing point between water of NC and UD  Downstream of NC prior Western Harbour |

**Table S2**

**Results of the paired t-test of differences of the environmental and nutrients between the two successive periods after three and eight years of insulation of wastewater effluents away from MB.**

| **Paired Samples Statistics** | | | | | **Paired Differences** | | | | | **t** | **df** | **Sig.**  **(2-tailed)** | **Percentage of**  **loss (-) and**  **gain (+)**  **in 2018 from 2013** |
| --- | --- | --- | --- | --- | --- | --- | --- | --- | --- | --- | --- | --- | --- |
| **Pairs (Parameters)** | **Year** | **Mean** | **Std. Deviation** | **Std. Error Mean** | **Mean** | **Std. Deviation** | **Std. Error Mean** | **95% Confidence Interval of the Difference** | |  |  |  |  |
|  |  |  |  |  |  |  |  | **Lower** | **Upper** |  |  |  |  |
| **TD** | 2018 | 176 | 116 | 27 | -42 | 79 | 19 | -80.97 | -2.36 | -2.24 | 17 | **0.04** | **-19** |
|  | 2013 | 217 | 165 | 39 |  |  |  |  |  |  |  |  |  |
| **STD** | 2018 | 49 | 20 | 5 | -6 | 30 | 7 | -20.51 | 8.85 | -0.84 | 17 | 0.41 | -11 |
|  | 2013 | 55 | 34 | 8 |  |  |  |  |  |  |  |  |  |
| **STD/TD** | 2018 | 39 | 27 | 6 | 8 | 30 | 7 | -6.80 | 23.35 | 1.16 | 17 | 0.26 | 27 |
|  | 2013 | 31 | 23 | 5 |  |  |  |  |  |  |  |  |  |
| **Water temperature** | 2018 | 24.6 | 4.2 | 1.0 | 1.3 | 2.8 | 0.7 | -0.03 | 2.72 | 2.06 | 17 | 0.05 | 6 |
|  | 2013 | 23.2 | 5.8 | 1.4 |  |  |  |  |  |  |  |  |  |
| **pH** | 2018 | 7.47 | 0.27 | 0.06 | -0.14 | 0.40 | 0.09 | -0.34 | 0.06 | -1.50 | 17 | 0.15 | -2 |
|  | 2013 | 7.61 | 0.48 | 0.11 |  |  |  |  |  |  |  |  |  |
| **Salinity** | 2018 | 2.70 | 0.94 | 0.22 | 0.06 | 0.51 | 0.12 | -0.20 | 0.31 | 0.48 | 17 | 0.64 | 2 |
|  | 2013 | 2.64 | 0.60 | 0.14 |  |  |  |  |  |  |  |  |  |
| **DO** | 2018 | 1.76 | 2.38 | 0.56 | -8.95 | 13.44 | 3.17 | -15.63 | -2.26 | -2.82 | 17 | **0.01** | **-84** |
|  | 2013 | 10.70 | 13.90 | 3.28 |  |  |  |  |  |  |  |  |  |
| **H_2_S** | 2018 | 4.11 | 7.10 | 1.67 | -26.92 | 43.91 | 10.35 | -48.76 | -5.09 | -2.60 | 17 | **0.02** | **-87** |
|  | 2013 | 31.03 | 46.88 | 11.05 |  |  |  |  |  |  |  |  |  |
| **Chlor-a** | 2018 | 38.21 | 45.56 | 10.74 | -39.60 | 99.04 | 23.34 | -88.85 | 9.65 | -1.70 | 17 | 0.11 | -51 |
|  | 2013 | 77.81 | 106.52 | 25.11 |  |  |  |  |  |  |  |  |  |
| **NH_4_** | 2018 | 0.219 | 0.213 | 0.050 | 0.03 | 0.23 | 0.05 | -0.09 | 0.14 | 0.50 | 17 | 0.62 | 14 |
|  | 2013 | 0.192 | 0.210 | 0.049 |  |  |  |  |  |  |  |  |  |
| **NO_2_** | 2018 | 0.010 | 0.011 | 0.003 | -0.01 | 0.02 | 0.00 | -0.02 | 0.00 | -1.65 | 17 | 0.12 | -40 |
|  | 2013 | 0.017 | 0.021 | 0.005 |  |  |  |  |  |  |  |  |  |
| **NO_3_** | 2018 | 0.034 | 0.038 | 0.009 | 0.01 | 0.04 | 0.01 | -0.01 | 0.03 | 0.68 | 17 | 0.51 | 24 |
|  | 2013 | 0.027 | 0.032 | 0.007 |  |  |  |  |  |  |  |  |  |
| **DIN** | 2018 | 0.263 | 0.204 | 0.048 | 0.03 | 0.24 | 0.06 | -0.09 | 0.15 | 0.48 | 17 | 0.64 | 11 |
|  | 2013 | 0.236 | 0.182 | 0.043 |  |  |  |  |  |  |  |  |  |
| **TN** | 2018 | 0.773 | 0.444 | 0.105 | -0.60 | 1.04 | 0.25 | -1.12 | -0.09 | -2.46 | 17 | **0.02** | **-44** |
|  | 2013 | 1.376 | 1.283 | 0.302 |  |  |  |  |  |  |  |  |  |
| **DIP** | 2018 | 0.019 | 0.015 | 0.004 | -0.01 | 0.01 | 0.00 | -0.01 | 0.00 | -1.91 | 17 | 0.07 | -25 |
|  | 2013 | 0.026 | 0.020 | 0.005 |  |  |  |  |  |  |  |  |  |
| **TP** | 2018 | 0.036 | 0.023 | 0.006 | -0.01 | 0.02 | 0.01 | -0.02 | 0.00 | -1.59 | 17 | 0.13 | -20 |
|  | 2013 | 0.045 | 0.029 | 0.007 |  |  |  |  |  |  |  |  |  |
